# Supplementary material for: Biotechnological Key Genes of the Rhodococcus erythropolis MGMM8 Genome: Genes for Bioremediation, Antibiotics, Plant Protection, and Growth Stimulation
Source: Microorganisms. 2023 Dec 31;12(1):88. doi: 10.3390/microorganisms12010088 (PMC10819586; doi:10.3390/microorganisms12010088)
Supplement: Supplementary file 1 [file microorganisms-12-00088-s001.zip › microorganisms-2727551-supplementary.pdf]

Biotechnological key genes of *Rhodococcus erythropolis* MGMM8 genome: Genes for bioremediation, antibiotics, plant protection, and growth stimulation

Daniel Mawuena Afordoanyi[1, 2*], Yaw Abayie Akosah[3*], Lidiya Shnakhova[4], Keremli Saparmyradov[1] Elena Shulga[1] ,Roderic Gilles Claret Diabankana[1] and Shamil Validov [1]

1 Laboratory of Molecular Genetics and Microbiology Methods, Kazan Scientific Center of Russian Academy of Sciences, 420111 Kazan, Russia.
2 Tatar Scientific Research Institute of Agricultural Chemistry and Soil Science, FRC Kazan Scientific Center, Russian Academy of Sciences, 420111 Kazan, Russia.
3 Department of Molecular Pathobiology, New York University College of Dentistry, New York, NY 10010, USA.
4 Dermatology Department, I.M. Sechenov First Moscow State Medical University (Sechenov University), Moscow, Russia.
* Correspondence: d.afordoanyi@knc.ru; r.diabankana@knc.ru

A. MGMM8

antiSMASH version 7.0.1

Download | About | Help | Contact

Select genomic region:

Overview | 1.1 | 1.2 | 1.3 | 1.4 | 1.5 | 1.6 | 1.7 | 1.8 | 1.9 | 1.10 | 1.11 | 1.12 | 1.13 | 1.14 | 1.15 | 1.16 | 1.17 | 1.18 | 1.19

Identified secondary metabolite regions using strictness 'relaxed'

CP124545.2 (Rhodococcus erythropolis)

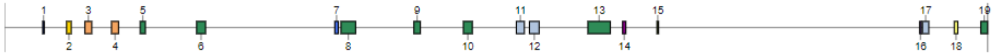

Region	Type	From	To	Most similar known cluster		Similarity
Region 1	RiPP-like	248,021	258,169	branched-chain fatty acids	Other	75%
Region 2	LAP	402,826	432,933	diisonitrile antibiotic SF2768	NRP	11%
Region 3	T1PKS	521,710	566,112			
Region 4	T1PKS	694,147	740,257	fulvuthiacene A/fulvuthiacene B	NRP+Polyketide	8%
Region 5	NAPAA	880,082	913,975	ε-Poly-L-lysine	NRP	100%
Region 6	NRP-metallophore , NRPS	1,246,203	1,304,246	erythrochelin	NRP	57%
Region 7	redox-cofactor	2,142,460	2,165,275	tetronasin	Polyketide	3%
Region 8	NRPS	2,182,512	2,277,514	corynecin III/corynecin I/corynecin II	Other	100%
Region 9	NRPS-like	2,655,310	2,697,405	pyrroloformamide A/pyrroloformamide B/pyrroloformamide D/pyrroloformamide C	NRP+Other	8%
Region 10	NRP-metallophore , NRPS	2,976,431	3,034,367	heterobactin A/heterobactin S2	NRP	100%
Region 11	NRPS , terpene	3,320,109	3,371,816	SF2575	Polyketide:Type II polyketide+Saccharide:Hybrid/tailoring saccharide	6%
Region 12	NRPS , RRE-containing	3,404,896	3,467,755	coelichelin	NRP	27%
Region 13	NRPS	3,784,130	3,929,608	monensin	Polyketide	5%
Region 14	terpene	4,009,093	4,030,037	carotenoid	Terpene	27%
Region 15	ectoine	4,231,984	4,242,382	ectoine	Other:Ectoine	75%
Region 16	butyrolactone	5,935,732	5,946,619			
Region 17	PKS-like , amglyccycl	5,954,029	5,995,057	acarbose	Saccharide	7%
Region 18	lanthipeptide-class-iii	6,160,641	6,183,223			
Region 19	NRPS	6,332,550	6,376,501			

B. JCM2895

antiSMASH version 7.0.1 | Download | About | Help | Contact

Select genomic region:

Overview | 1.1 | 1.2 | 1.3 | 1.4 | 1.5 | 1.6 | 1.7 | 1.8 | 1.9 | 1.10 | 1.11 | 1.12 | 1.13 | 1.14 | 1.15 | 1.16 | 1.17 | 1.18 | 1.19

Identified secondary metabolite regions using strictness 'relaxed'

NZ_AP018733.1 (Rhodococcus erythropolis)

Region	Type	From	To	Most similar known cluster		Similarity
Region 1	LAP	125,965	156,061	diisonitrile antibiotic SF2768	NRP	11%
Region 2	T1PKS	245,004	289,409			
Region 3	T1PKS	410,926	457,036	fulvuthiacene A/fulvuthiacene B	NRP+Polyketide	8%
Region 4	NAPAA	595,757	629,650	ε-Poly-L-lysine	NRP	100%
Region 5	NRP-metallophore, NRPS	969,491	1,027,543	erythrochelin	NRP	57%
Region 6	redox-cofactor	1,857,852	1,880,667	tetronasin	Polyketide	3%
Region 7	NRPS	1,899,383	2,010,321	corynecin III/corynecin I/corynecin II	Other	100%
Region 8	NRPS-like	2,399,895	2,442,147	pyrroloformamide A/pyrroloformamide B/pyrroloformamide D/pyrroloformamide C	NRP+Other	8%
Region 9	NRP-metallophore, NRPS	2,750,896	2,808,836	heterobactin B/heterobactin S2	NRP	100%
Region 10	NRPS, terpene	3,097,081	3,150,209	SF2575	Polyketide:Type II polyketide+Saccharide:Hybrid/tailoring saccharide	6%
Region 11	NRPS, RRE-containing	3,183,440	3,246,339	coelichelin	NRP	27%
Region 12	NRPS	3,482,803	3,548,199	rifamorpholine A/rifamorpholine B/rifamorpholine C/rifamorpholine D/rifamorpholine E	Polyketide	3%
Region 13	NRPS	3,551,927	3,606,582	monensin	Polyketide	5%
Region 14	terpene	3,686,525	3,707,469	carotenoid	Terpene	27%
Region 15	ectoine	3,910,168	3,920,566	ectoine	Other:Ectoine	75%
Region 16	butyrolactone	5,621,464	5,632,351			
Region 17	lanthipeptide-class-iii	5,880,331	5,902,913			
Region 18	NRPS	6,262,069	6,306,475	polyoxin A/polyoxin H	Other	5%
Region 19	RiPP-like	6,391,794	6,403,724	branched-chain fatty acids	Other	75%

C. X5

antiSMASH version 7.0.1 — Download — About — Help — Contact

Select genomic region:

Overview | 1.1 | 1.2 | 1.3 | 1.4 | 1.5 | 1.6 | 1.7 | 1.8 | 1.9 | 1.10 | 1.11 | 1.12 | 1.13 | 1.14 | 1.15 | 1.16 | 1.17 | 1.18

Identified secondary metabolite regions using strictness 'relaxed'

NZ_CP044284.1 (Rhodococcus erythropolis)

Region	Type	From	To	Most similar known cluster		Similarity
Region 1	LAP	116,838	146,934	diisonitrile antibiotic SF2768	NRP	11%
Region 2	T1PKS	236,157	280,586			
Region 3	T1PKS	420,459	466,569	fulvuthiacene A/fulvuthiacene B	NRP+Polyketide	8%
Region 4	NAPAA	606,766	640,659	ε-Poly-L-lysine	NRP	100%
Region 5	NRP-metallophore, NRPS	980,500	1,038,534	erythrochelin	NRP	57%
Region 6	redox-cofactor	1,838,993	1,861,808	tetronasin	Polyketide	3%
Region 7	NRPS	1,880,524	1,988,279	corynecin III/corynecin I/corynecin II	Other	100%
Region 8	NRPS-like	2,450,634	2,492,427			
Region 9	NRP-metallophore, NRPS	2,858,916	2,916,856	heterobactin B/heterobactin S2	NRP	100%
Region 10	NRPS, terpene	3,205,049	3,258,029	SF2575	Polyketide:Type II polyketide+Saccharide:Hybrid/tailoring saccharide	6%
Region 11	NRPS, RRE-containing	3,291,256	3,354,129	coelichelin	NRP	27%
Region 12	NRPS	3,590,593	3,655,989	rifamorpholine A/rifamorpholine B/rifamorpholine C/rifamorpholine D/rifamorpholine E	Polyketide	3%
Region 13	NRPS	3,658,911	3,715,122	monensin	Polyketide	5%
Region 14	terpene	3,794,315	3,815,259	carotenoid	Terpene	27%
Region 15	ectoine	4,017,958	4,028,356	ectoine	Other:Ectoine	75%
Region 16	butyrolactone	5,766,086	5,776,973			
Region 17	lanthipeptide-class-iii	6,006,250	6,028,832			
Region 18	RiPP-like	6,415,275	6,427,205	branched-chain fatty acids	Other	75%

D. D310-1

antiSMASH version 7.0.1

Download About Help Contact

Select genomic region:

Overview 1.1 1.2 1.3 1.4 1.5 1.6 1.7 1.8 1.9 1.10 1.11 1.12 1.13 1.14 1.15 1.16 1.17 1.18

Identified secondary metabolite regions using strictness 'relaxed'

NZ_CP032403.1 (Rhodococcus erythropolis)

Region	Type	From	To	Most similar known cluster		Similarity
Region 1	LAP	102,825	132,918	diisonitrile antibiotic SF2768	NRP	11%
Region 2	T1PKS	223,958	268,377			
Region 3	T1PKS	415,858	461,959	fulvuthiacene A/fulvuthiacene B	NRP+Polyketide	8%
Region 4	NAPAA	605,284	639,177	ε-Poly-L-lysine	NRP	100%
Region 5	NRP-metallophore, NRPS	988,683	1,046,654	erythrochelin	NRP	57%
Region 6	redox-cofactor	1,864,380	1,887,195	tetronasin	Polyketide	3%
Region 7	NRPS	1,904,978	2,009,465	corynecin III/corynecin I/corynecin II	Other	100%
Region 8	NRPS-like	2,433,838	2,475,882			
Region 9	NRP-metallophore, NRPS	2,773,975	2,831,891	heterobactin A/heterobactin S2	NRP	100%
Region 10	NRPS, terpene	3,122,623	3,176,030	SF2575	Polyketide:Type II polyketide+Saccharide:Hybrid/tailoring saccharide	6%
Region 11	NRPS, RRE-containing	3,208,672	3,273,198	coelichelin	NRP	27%
Region 12	NRPS	3,515,691	3,684,637	rifamorpholine A/rifamorpholine B/rifamorpholine C/rifamorpholine D/rifamorpholine E	Polyketide	4%
Region 13	terpene	3,763,591	3,784,535	carotenoid	Terpene	27%
Region 14	ectoine	3,996,913	4,007,311	ectoine	Other:Ectoine	75%
Region 15	butyrolactone	5,745,795	5,756,682			
Region 16	PKS-like, amglyccycl	5,781,938	5,822,966	acarbose	Saccharide	7%
Region 17	lanthipeptide-class-iii	5,996,350	6,018,932			
Region 18	RiPP-like	6,457,414	6,469,344	branched-chain fatty acids	Other	75%

E. CCM2595

antiSMASH version 7.0.1 Download About Help

Select genomic region:

Overview 1.1 1.2 1.3 1.4 1.5 1.6 1.7 1.8 1.9 1.10 1.11 1.12 1.13 1.14 1.15 1.16 1.17 1.18

Identified secondary metabolite regions using strictness 'relaxed'

NC_022115.1 (Rhodococcus erythropolis CCM2595)

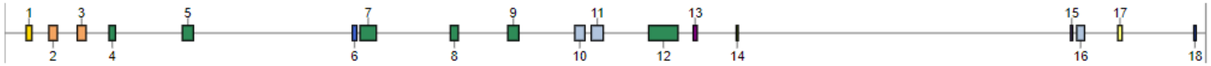

Region	Type	From	To	Most similar known cluster		Similarity
Region 1	LAP	113,035	143,139	diisonitrile antibiotic SF2768	NRP	11%
Region 2	T1PKS	231,918	276,327			
Region 3	T1PKS	380,348	426,458	fulvuthiacene A/fulvuthiacene B	NRP+Polyketide	8%
Region 4	NAPAA	546,300	580,193	ε-Poly-L-lysine	NRP	100%
Region 5	NRP-metallophore , NRPS	929,846	987,813	erythrochelin	NRP	57%
Region 6	redox-cofactor	1,818,682	1,841,497	tetronasin	Polyketide	3%
Region 7	NRPS	1,858,862	1,944,237	corynecin III/corynecin I/corynecin II	Other	100%
Region 8	NRPS-like	2,331,159	2,372,500			
Region 9	NRP-metallophore , NRPS	2,631,307	2,689,241	heterobactin B/heterobactin S2	NRP	100%
Region 10	NRPS , terpene	2,981,614	3,034,493	SF2575	Polyketide:Type II polyketide+Saccharide:Hybrid/tailoring saccharide	6%
Region 11	NRPS , RRE-containing	3,067,661	3,130,551	coelichelin	NRP	27%
Region 12	NRPS	3,367,855	3,521,396	monensin	Polyketide	5%
Region 13	terpene	3,600,687	3,621,631	isorenieratene	Terpene	37%
Region 14	ectoine	3,825,710	3,836,108	ectoine	Other:Ectoine	75%
Region 15	butyrolactone	5,574,079	5,584,966			
Region 16	PKS-like , amglyccycl	5,605,866	5,646,894	acarbose	Saccharide	7%
Region 17	lanthipeptide-class-iii	5,820,723	5,843,305			
Region 18	RiPP-like	6,218,252	6,230,182	branched-chain fatty acids	Other	75%

Figure S1. AntiSMASH results for the prediction of secondary metabolites cluster genes of *R. erythropolis* MGMM8 (A), JCM2895 (B), X5(C), D310-1(D), and CCM2595 (E) core genomes.

A. MGMM

CARD: RGI Results

Back to RGI

Download Results

CP124545.2.fasta

Table View | AMR Genes | AMR Gene Family | Drug Class | Resistance Mechanism

Summary (summary counts and figures only include Loose hits of e-10 or better)

Filename	Date (UTC)	RGI Criteria	# Perfect Hits	# Strict Hits	# Loose Hits	Download
CP124545.2	September 04, 2023 14:34:59	Perfect, Strict, complete genes only	0	4	0	Download

Results (all Loose hits shown)

Search:

RGI Criteria	ARO Term	SNP	Detection Criteria	AMR Gene Family	Drug Class	Resistance Mechanism	% Identity of Matching Region	% Length of Reference Sequence
Strict	vanW gene in vanI cluster		protein homolog model	vanW, glycopeptide resistance gene cluster	glycopeptide antibiotic	antibiotic target alteration	29.74	205.36
Strict	vanY gene in vanB cluster		protein homolog model	vanY, glycopeptide resistance gene cluster	glycopeptide antibiotic	antibiotic target alteration	29.92	72.39
Strict	iri		protein homolog model	rifampin monooxygenase	rifamycin antibiotic	antibiotic inactivation	95.56	99.16
Strict	RbpA		protein homolog model	RbpA bacterial RNA polymerase-binding protein	rifamycin antibiotic	antibiotic target protection	85.59	98.25
Strict	Mycobacterium tuberculosis folC with mutation conferring resistance to para-aminosalicylic acid	L56V	protein variant model	aminosalicylate resistant dihydrofolate synthase	salicylic acid antibiotic	antibiotic target alteration	67.77	98.36

B. JCM 2895

CARD: RGI Results

← Back to RGI

Download Results

NZ_AP018733.1.fasta
CP124545.2.fasta

Table View | AMR Genes | AMR Gene Family | Drug Class | Resistance Mechanism

Summary (summary counts and figures only include Loose hits of e-10 or better)

Filename	Date (UTC)	RGI Criteria	# Perfect Hits	# Strict Hits	# Loose Hits	Download
NZ_AP018733.1	September 04, 2023 14:35:41	Perfect, Strict, complete genes only	0	4	0	Download

Results (all Loose hits shown)

Search:

RGI Criteria	ARO Term	SNP	Detection Criteria	AMR Gene Family	Drug Class	Resistance Mechanism	% Identity of Matching Region	% Length of Reference Sequence
Strict	vanW gene in vanI cluster		protein homolog model	vanW, glycopeptide resistance gene cluster	glycopeptide antibiotic	antibiotic target alteration	29.74	203.75
Strict	Streptomyces venezuelae rox		protein homolog model	rifampin monooxygenase	rifamycin antibiotic	antibiotic inactivation	66.18	100.42
Strict	RbpA		protein homolog model	RbpA bacterial RNA polymerase-binding protein	rifamycin antibiotic	antibiotic target protection	86.49	98.25
Strict	Mycobacterium tuberculosis folC with mutation conferring resistance to para-aminosalicylic acid	L56V	protein variant model	aminosalicylate resistant dihydrofolate synthase	salicylic acid antibiotic	antibiotic target alteration	67.77	98.36

C. X5

CARD: RGI Results

Back to RGI

Download Results

NZ_CP044284.1.fasta
NZ_AP018733.1.fasta
CP124545.2.fasta

Table View | AMR Genes | AMR Gene Family | Drug Class | Resistance Mechanism

Summary (summary counts and figures only include Loose hits of e-10 or better)

Filename	Date (UTC)	RGI Criteria	# Perfect Hits	# Strict Hits	# Loose Hits	Download
NZ_CP044284.1	September 04, 2023 14:36:34	Perfect, Strict, complete genes only	0	4	0	Download

Results (all Loose hits shown)

Search:

RGI Criteria	ARO Term	SNP	Detection Criteria	AMR Gene Family	Drug Class	Resistance Mechanism	% Identity of Matching Region	% Length of Reference Sequence
Strict	vanW gene in vanI cluster		protein homolog model	vanW, glycopeptide resistance gene cluster	glycopeptide antibiotic	antibiotic target alteration	29.74	203.75
Strict	Streptomyces venezuelae rox		protein homolog model	rifampin monooxygenase	rifamycin antibiotic	antibiotic inactivation	66.18	100.42
Strict	RbpA		protein homolog model	RbpA bacterial RNA polymerase-binding protein	rifamycin antibiotic	antibiotic target protection	86.49	98.25
Strict	Mycobacterium tuberculosis folC with mutation conferring resistance to para-aminosalicylic acid	L56V	protein variant model	aminosalicylate resistant dihydrofolate synthase	salicylic acid antibiotic	antibiotic target alteration	67.77	98.36

D. D310-1

CARD: RGI Results

Back to RGI

Download Results

NZ_CP032403.1.fasta
NZ_CP044284.1.fasta
NZ_AP018733.1.fasta
CP124545.2.fasta

Table View | AMR Genes | AMR Gene Family | Drug Class | Resistance Mechanism

Summary (summary counts and figures only include Loose hits of e-10 or better)

Filename	Date (UTC)	RGI Criteria	# Perfect Hits	# Strict Hits	# Loose Hits	Download
NZ_CP032403.1	September 04, 2023 14:37:40	Perfect, Strict, complete genes only	0	4	0	Download

Results (all Loose hits shown)

Search:

RGI Criteria	ARO Term	SNP	Detection Criteria	AMR Gene Family	Drug Class	Resistance Mechanism	% Identity of Matching Region	% Length of Reference Sequence
Strict	vanW gene in vanI cluster		protein homolog model	vanW, glycopeptide resistance gene cluster	glycopeptide antibiotic	antibiotic target alteration	30.17	200.54
Strict	Streptomyces venezuelae rox		protein homolog model	rifampin monooxygenase	rifamycin antibiotic	antibiotic inactivation	67.09	104.20
Strict	RbpA		protein homolog model	RbpA bacterial RNA polymerase-binding protein	rifamycin antibiotic	antibiotic target protection	86.49	98.25
Strict	Mycobacterium tuberculosis folC with mutation conferring resistance to para-aminosalicylic acid	L56V	protein variant model	aminosalicylate resistant dihydrofolate synthase	salicylic acid antibiotic	antibiotic target alteration	67.56	98.36

E. CCM2595

CARD: RGI Results

Back to RGI

Download Results

NC_022115.1.fasta

CP124545.2.fasta

Table View | AMR Genes | AMR Gene Family | Drug Class | Resistance Mechanism

Summary (summary counts and figures only include Loose hits of e-10 or better)

Filename	Date (UTC)	RGI Criteria	# Perfect Hits	# Strict Hits	# Loose Hits	Download
NC_022115.1	September 04, 2023 14:39:16	Perfect, Strict, complete genes only	0	4	0	Download

Results (all Loose hits shown)

Search:

RGI Criteria	ARO Term	SNP	Detection Criteria	AMR Gene Family	Drug Class	Resistance Mechanism	% Identity of Matching Region	% Length of Reference Sequence
Strict	vanW gene in vanI cluster		protein homolog model	vanW, glycopeptide resistance gene cluster	glycopeptide antibiotic	antibiotic target alteration	29.74	203.75
Strict	vanY gene in vanB cluster		protein homolog model	vanY, glycopeptide resistance gene cluster	glycopeptide antibiotic	antibiotic target alteration	29.92	72.39
Strict	iri		protein homolog model	rifampin monooxygenase	rifamycin antibiotic	antibiotic inactivation	96.19	99.16
Strict	RbpA		protein homolog model	RbpA bacterial RNA polymerase-binding protein	rifamycin antibiotic	antibiotic target protection	85.59	98.25
Strict	Mycobacterium tuberculosis folC with mutation conferring resistance to para-aminosalicylic acid	L56V	protein variant model	aminosalicylate resistant dihydrofolate synthase	salicylic acid antibiotic	antibiotic target alteration	67.77	98.36

Figure S2. Antibiotic resistance genes harbored in the core genomes of *R. erythropolis* MGMM8 (A), JCM2895 (B), X5 (C), D310-1 (D), CCM2595 (E).

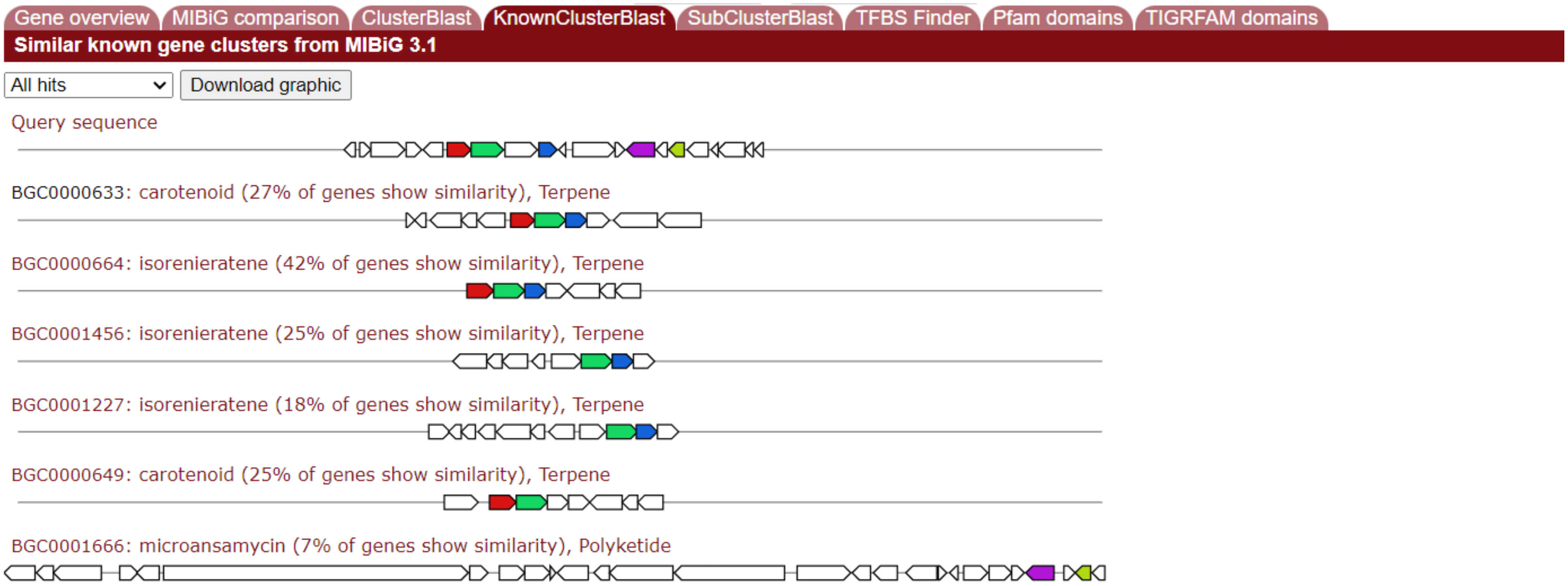

Figure S3. Percentage identity on the extra features of the KnownClusterBlast feature for Terpene type carotenoid on antiSMAH 7.0.1

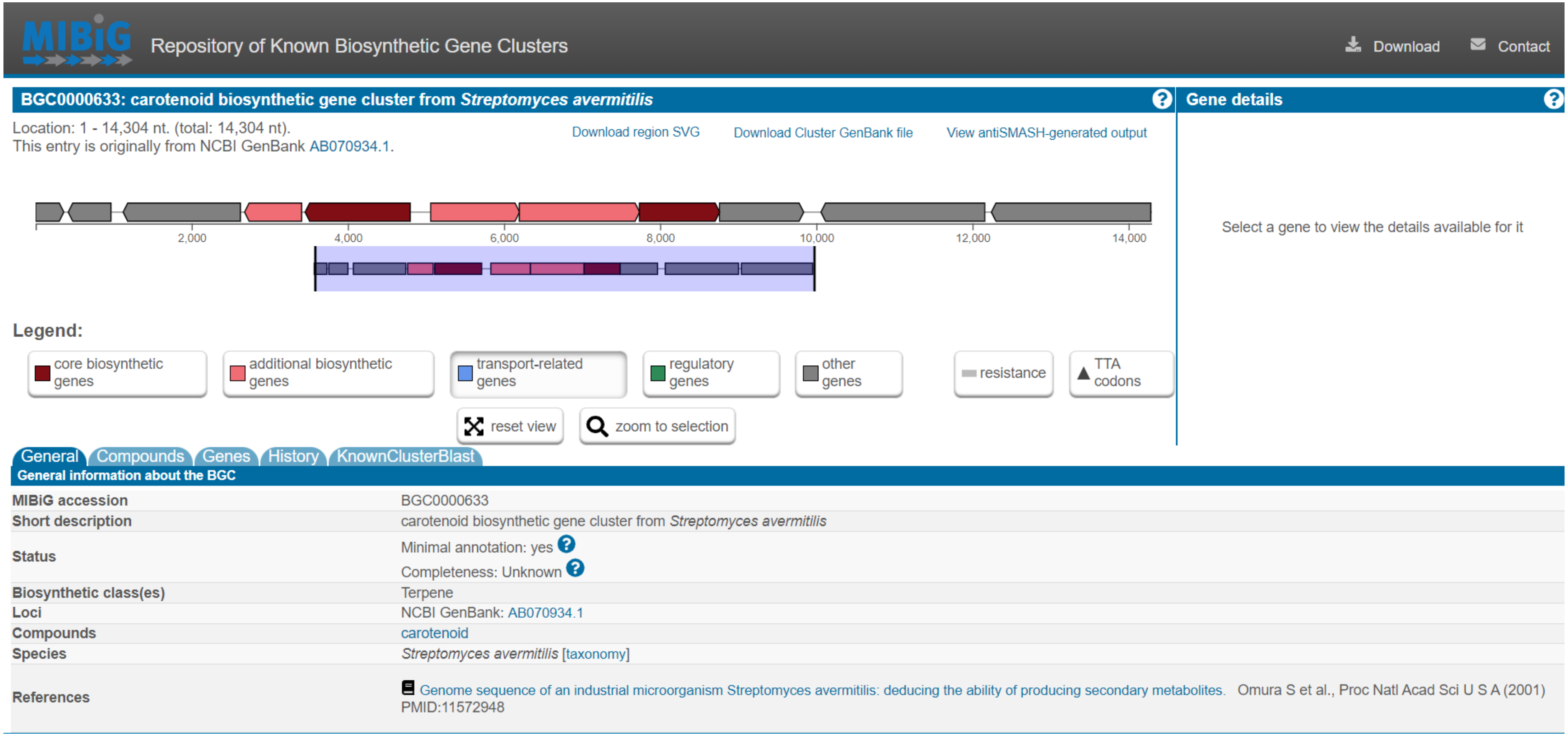

Figure S4. Identified biosynthetic gene cluster of *Streptomyces avermitilis* Terpene type carotenoid the Minimum Information about a Biosynthetic Gene cluster (MIBiG) database predicted for *R. erythtropolis* MGMM8 on the antiSMAH 7.0.1 server.

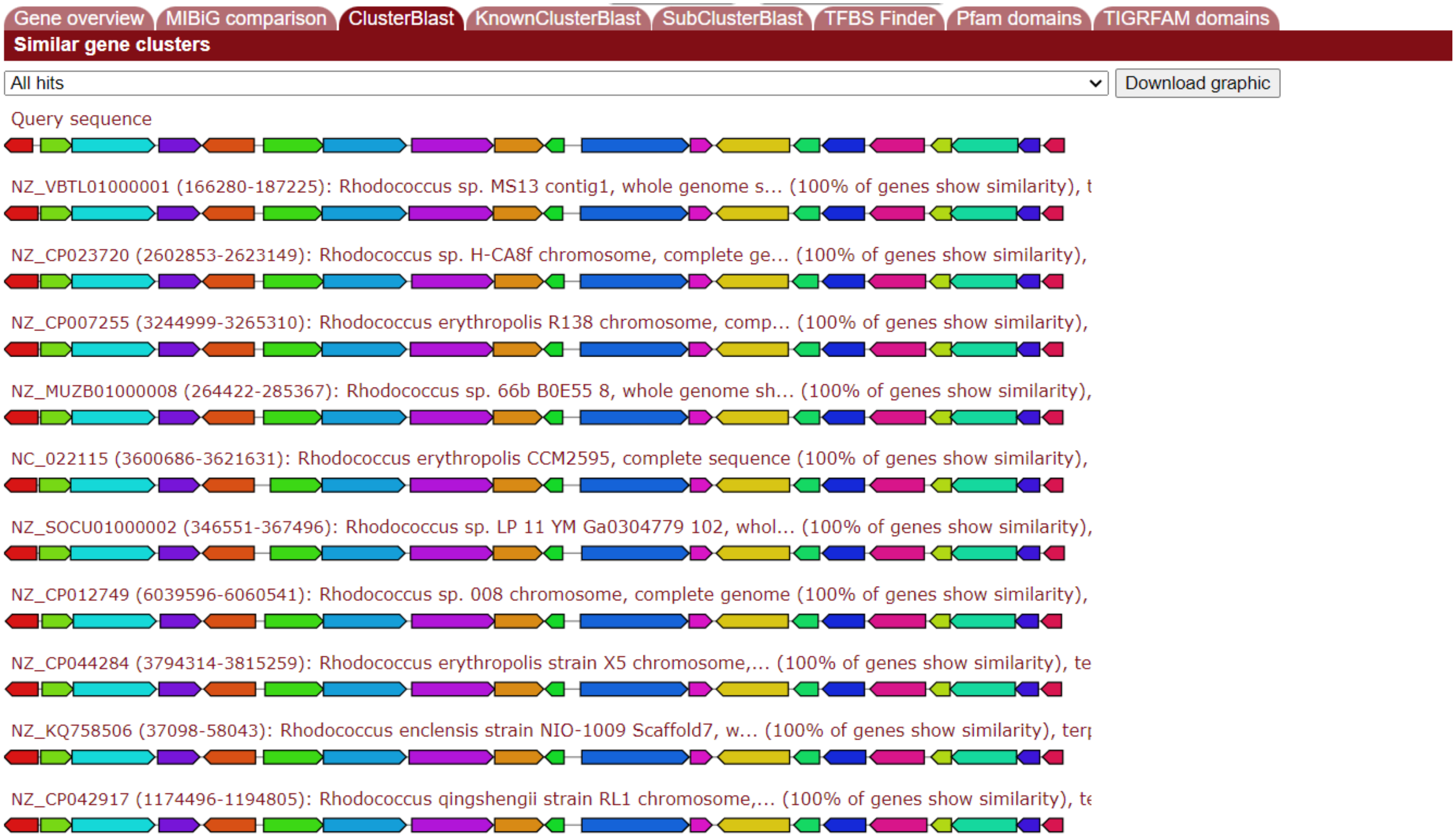

Figure S5. Percentage identity for the ClusterBlast feature for Terpene type carotenoid on antiSMAH 7.0.1 with 100% identity to strains of *Rhodococcus* spp.
